# Anais Brasileiros de Dermatologia: updating guidelines for authors

**DOI:** 10.1016/j.abd.2022.10.001

**Published:** 2022-11-17

**Authors:** Sílvio Alencar Marques, Ana Maria Roselino, Hiram Laranjeira de Almeida, Luciana P. Fernandes Abbade

**Affiliations:** a Universidade Estadual Paulista, Botucatu, SP, Brazil; b Universidade de São Paulo, Ribeirão Preto, SP, Brazil; c Universidade Federal de Pelotas, Pelotas, RS, Brazil; d Universidade Católica de Pelotas, Pelotas, RS, Brazil

Dear colleagues,

To keep pace with the dynamism of the evolution of medical knowledge, the journal that transmits it must be equally dynamic and, as an example, prioritize articles that deal with emerging diseases, such as monkeypox, adverse events, or the impacts of anti-SARS-CoV2 vaccines. or even endemic diseases that show high sanitary impact.^1^, ^2^, ^3^ Faithful to this premise, the norms of the Anais Brasileiros de Dermatologia (ABD) have been updated aiming to speed up, value and improve the transmission of dermatological knowledge and make it more attractive to authors and readers. Specific sections such as “Images” and “What's your diagnosis?” cease to exist, because, in fact, they are clinical cases that can be disclosed in a more succinct and objective way. Likewise, the section “Tropical/Infectoparasitic Dermatology” has been transferred to the letter section, maintaining its emphasis and specific focus, thus becoming “Tropical/Infectoparasitic Dermatology - Letter”. Likewise, “Dermatopathology” has migrated and is now included as “Dermatopathology - Letter”. The subsection “Therapy - Letter” has also been created, which opens up a specific space for clinical cases in which emphasis is on results obtained with new drugs, or for the communication of possible adverse events of greater relevance. Moreover, the communication of clinical cases of upmost interest is maintained under the name “Case Letter”. Besides, it is important to point out that the “Letter” section has standardized rules regardless of subtype. “Abstract” and “Keywords” are no longer necessary and the number of authors is limited to six, with four figures, 10 references, and 700 words, except for the “Research Letter” which maintains the 1000-word limit. The authors define which subsection of “Letters” best fits their article and will find specific formatting norms in the Elsevier submission system.

In addition to the important structural change described above, there are adjustments to the text of the other sections, guidelines, and useful links to help authors. The link to a checklist of summarized reports on each section and its rules, specifications regarding images, tables, and requirements regarding documentation is highlighted.

We remain fully committed to the quality and dissemination of ABD, not only within the national and Ibero-Latin American scientific community but also in the dermatological academic world as a whole. We move forward with the common objective of reporting the best science, for the improvement of dermatology, and, as a result, the best care for the patient. Come along, ABD reader, and be a part of this effort.

## Conflicts of interest

None declared.
